# Movement of leopard tortoises in response to environmental and climatic variables in a semi-arid environment

**DOI:** 10.1186/s40462-017-0096-y

**Published:** 2017-03-20

**Authors:** Martyn Drabik-Hamshare, Colleen T. Downs

**Affiliations:** School of Life Sciences, University of KwaZulu-Natal, Private Bag X01, Scottsville, Pietermaritzburg, KwaZulu-Natal 3209 South Africa

**Keywords:** Spatial ecology, Water loss, Karoo, *Stigmochelys pardalis*, Environmental variables, Electric fencing

## Abstract

**Background:**

Tortoises (Testudinidae) occur in a wide range of environments, providing important ecosystem functions, such as seed dispersal and refuge in the form of burrows. Tortoise movement has previously been shown to be related to resource availability, reproductive status and local environmental conditions. However, understanding of the variables that drive their movement remains comparatively low. We investigated aspects of movement in leopard tortoises *Stigmochelys pardalis*—the largest and most abundant tortoise species in sub-Saharan Africa—in response to environmental, climatic and individual variables in the semi-arid Karoo, South Africa. We used GPS telemetry to calculate bihourly and daily movement and used generalized linear mixed models (GLMMs) to ascertain important predictor variables.

**Results:**

Temperature, distance from water sources, and month were important variables for predicting both bihourly and daily movement. Our results showed that movement increased when individuals were close to known water sources, indicating that individuals close to water resources make regular long distance movements. Movement showed a positive relationship for temperature in both models, whilst rainfall was an important predictor for bihourly movement. Our results displayed aspects of seasonality, with movement highest in spring months, likely related to reproductive activities, although no sex differences were observed.

**Conclusions:**

We identified temporal and spatial conditions in which leopard tortoise movement increased. Our results further support the relationship between water as a resource and movement in leopard tortoises. Individuals used one of two basic movement behaviours in relation to water in this water scarce environment. Either an individual’s home range and movements included permanent water resources allowing internal water storage replenishment, or excluded these with reliance on food resources (such as grasses, forbs, and succulents) for water.

## Background

Continual growth of human population increases the need to harvest and distribute essential resources, causing modifications to environments, and subsequent disturbance and contamination of local ecosystems [1]. Such land use change is a primary cause for damage to ecosystems and animal populations [2], as it directly relates to habitat loss, habitat defragmentation, and global warming [3]. It is of great importance to conduct systematic research with regards to potential effects of land use change, in order to produce effective decision-making and management for protection and conservation of endangered and threatened species and habitats. Land use change in the Central Karoo over the last few centuries has greatly affected animal populations, with the vast majority of pre-existing lands now converted to private commercial farming. Introduction of livestock, building of roads and fences, and reliance of animal and human communities on already depleted water supplies, has negatively affected many animal and plant species. For example, wattled cranes (*Bugeranus carunculatus*), southern bald ibis (*Geronticus calvus*), and Cape vultures (*Gyps coprotheres*)—regionally common before the arrival of Europeans (*c*. 1650)—are now all but extinct regionally, partly due to changes in availability of water and natural food resources [4–6]. Changes in land use in the Karoo are expected to continue with the introduction of hydraulic fracturing (fracking) activities: a process whereby fuel is extracted from deep within the Earth’s surface following the injection of a highly pressurised liquid fluid [7]. Fracking operations are expected before end of 2017 [8, 9], despite worries about impacts on human and animal communities due to increasing water salinity and altering water quality through accidental release of water runoff [10, 11].

The Karoo is an important ecosystem, as it is seen as a centre for endemism in birds and reptiles [12, 13]. For example, of the 18 tortoise species in sub-Saharan Africa, at least eight species occur somewhere in the Karoo: up to five sympatrically [14–16]. Tortoises are of the most threatened animals, with as many as 80% classified at least as ‘Vulnerable’, and 47% at least as ‘Endangered’ by the International Union for the Conservation of Nature (IUCN) [14, 17]. The importance of tortoises to their environments is increasingly being understood. Tortoises provide an important ecosystem function in the form of seed dispersal [18, 19] promoted by periodical long distance movement and long gut retention time [20]. This function is particularly importance in xeric areas where natural herbivores are no longer present. Tortoises are considered keystone species in some regions. For example, burrowing species such as *Gopherus* spp. produce refugia used by multiple species to escape harsh environmental conditions [21]. It is important to improve understanding of tortoise spatial ecology.

Tortoises are able to tolerate imbalances in regards to their water:electrolyte ratio [22, 23], allowing a greater ability to survive drought conditions [23, 24]. However, drinking water remains necessary to facilitate urination to remove waste products, which otherwise can cause severe stress and mortality [24]. Several studies investigating spatial ecology of tortoises have identified the positive relationship between movement and water (e.g. permanent water sources or rainfall) with movement typically increased after periods of higher rainfall [22, 25–29]. Increased tortoise movement has also been related to higher temperatures [30, 31], seasonality (e.g. higher in spring) [31–34], and reproductive status (search for mates, egg-laying habitat and resources to feed increased energy demand) [32, 35–38]. Resource availability also appears to be of importance. For example, distribution and movement in Aldabra tortoises (*Aldabrachelys gigantea*) appears to be related to resources [39], whilst the Santa Cruz giant tortoise of the Galápagos archipelago (*Chelonoidis nigra*) undertakes seasonal altitudinal migrations in response to vegetation dynamics [40]. In contrast, most other tortoise species maintain home ranges, instead modifying home range size in response to resource availability [22, 34]. Further information is required to better understand interactions between tortoises and environmental conditions.

The leopard tortoise (*Stigmochelys pardalis*) is the largest tortoise species in sub-Saharan Africa, inhabiting a wide range of environmental conditions across the eastern and southern parts of the continent [14, 15]. The species is currently classified by IUCN as ‘Least Concern’ [14, 36], though they appear to be particularly vulnerable to electric fencing, which is common in Karoo farms to control predation on livestock by caracal (*Caracal caracal*) and black-backed jackal (*Canis mesomelas*) [41]. Previous leopard tortoise research has shown great variability in movement distances and home range sizes, likely related to seasonal temperature, food availability, rainfall, mean body mass, and access to other important resources [31, 33, 42]. For example, leopard tortoises were shown to move much larger distances in the Nama-Karoo (up to 8 km per day) [33]—even displaying nomadic behaviour in some cases [26]—when compared with populations in valley thicket (up to 100 m per day) [43] and Swaziland (about 50 m per day) [31]. Karoo leopard tortoises also have larger home range sizes, using areas upwards of 200 ha [33] compared with valley thicket (57.56 ha) and Swaziland (13.49 ha). These studies suggest that movement and home range is higher in areas where resource availability (e.g. food, water, and mates) is decreased. Despite several studies investigating movement of leopard tortoises, information on drivers of movement and habitat use is not fully understood.

Geolocation information helps to understand species interactions, identify important habitats, and quantify the relationship between behaviour and climatic and environmental variables [44]. Improving knowledge of spatial ecology is important to identify biotic and abiotic effects relating to land use, and to guide successful management decisions for species conservation [45]. Global positioning system (GPS) transmitters were deployed on ten wild-caught individuals. We set out to further investigate spatial ecology of leopard tortoises, to a) provide details on movement distances in relation to climatic, environmental and sex variables, and b) highlight importance of water and food resources.

We predicted higher movement closer to important resources (e.g. food and water), as previous studies have shown increased activity when resources are abundant [22]. We predicted climatic variables (temperature and rainfall) would positively influence movement, as higher temperatures causes increased metabolic rates; whilst tortoises are expected to seek natural water sources after rain events [22, 28]. Finally, we expected tortoises would make larger movements during the breeding season (September to November), as mate-searching, egg-laying, and associated increases in energy demand is increased [46, 47]. However, given leopard tortoises can occur at very low densities (0.017 tortoises per ha) [25], we expected mate-searching behaviour by males would produce higher movements overall.

## Methods

### Study area

The semi-arid Karoo covers much of the Northern, Western and Eastern Cape Provinces of South Africa, covering an area of approximately 37 million ha [48]. Northern and western parts of the Karoo are typically arid, though even in eastern semi-arid areas, rainfall is both unpredictable and unreliable [49, 50]. During summer, daily temperatures of more than 30 °C are regularly recorded [49], whilst severe frost events also occur [51]. Plants in the region have adapted to such conditions—hairy cuticles, tannins and phenolic compounds [52]—to cope with severe stress and desiccation [48, 49, 53]. Due to common weather conditions, vegetation of the Central Karoo is highly homogenous with typically low levels of endemism [54].

**Fig. 1:**
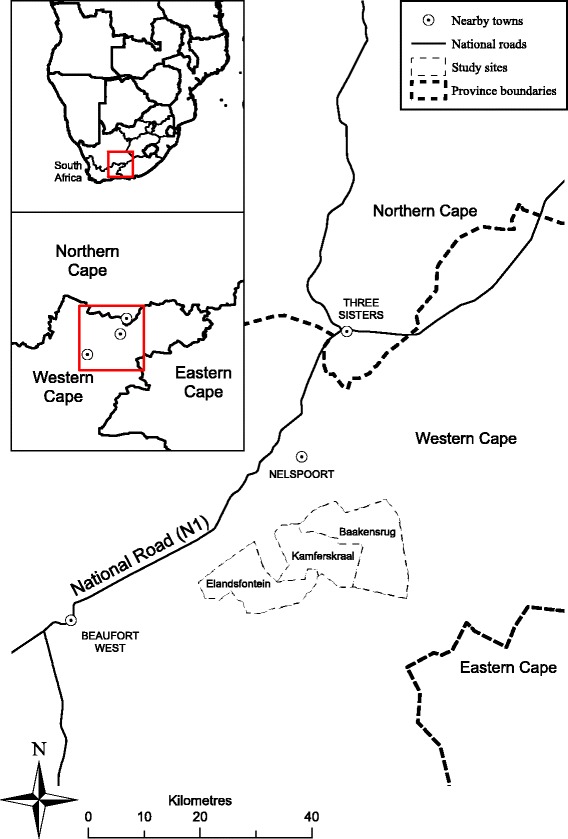
Study sites. Local area map of study sites near Beaufort West, South Africa

The study was carried out on three private mixed livestock commercial farms in the Central Karoo, Western Cape Province, South Africa (Fig. 1). The farms used were Baakensrug, Kamferskraal, and Elandsfontein (approximately 32°15S, 23°E), which are part of the Nelspoort and Beaufort West communities. Each farm utilises aspects of holistic resource management, with rotational grazing of mixed livestock to reduce selective grazing and subsequent desertification [55]. Private hunting of free-roaming game is also present. Whilst the three farms are connected, roads, fences and mountain ranges form distinct boundaries (unpublished observations). These farms use various agricultural fencing to separate pastures of varying sizes and protect livestock. These fences have varying levels of restriction and risk to tortoises; from little (e.g. low tensile wire fence) to full (e.g. chain-link fence). In some areas, farms also use electric fencing to prevent animals digging under agricultural fencing. These electric fences present a major mortality risk for tortoises [56, 57].

### Fieldwork

As tortoises generally have a bimodal activity pattern, especially in spring and summer [28, 58, 59], wild-caught adult leopard tortoises were located by walking 2 km transects (*n* = 20) in mornings and evenings in November and December 2014. Transect locations on farms were determined using random points in ArcGIS 10.3.1 (ESRI, CA, USA). Upon locating each individual, digital hanging scales (Pesola, Schindellegi, Switzerland) were used to measure body mass (g).

Unique GPS-Global System for Mobile Communications (GSM)/Ultra High Frequency (UHF) transmitters (Wireless Wildlife, Pretoria, South Africa) were initially placed on adult leopard tortoises (*n* = 10). Care was taken to avoid placing transmitters across scutes to avoid problems relating to growth. Tortoises were selected based on body mass (mean: 13.92 kg, range: 7.43 to 26.27 kg) and sex. We determined sex of individuals based on plastral concavity, tail length, and shapes of anal scutes and supracaudal shield [60, 61]. The transmitter was placed appropriately on the carapace to avoid inhibiting potential mating events (e.g. front of carapace for females) using dental acrylic. Mass of transmitters (74 g) was much lower (range: 0.3 to 1.0%) than the suggested 5% body mass [62]. Transmitters were programmed to receive bihourly geolocation data for a minimum period of 12 months, based on expected internal battery life. Individuals were released at initial point of location within 30 min.

In order to download telemetry data, the study area was revisited four times (approximately every 3 months) throughout 2015. A base-station was used to communicate with the transmitters to download internally-stored data. The base-station was positioned at high elevations, as direct ‘line-of-sight’ between base-station and transmitters was required. Once downloaded, the base-station sent data via a local cell-phone network. Raw telemetry data were downloaded as a CSV file via Wireless Wildlife [63]. On each visit, attempts were made to physically locate telemetered individuals to assess condition of each tortoise, using recently downloaded data. Whilst no body condition index was used, we assessed condition based on levels of activity, general well-being, and changes to body mass. In cases where individuals could not be physically located, condition was assessed based on recent movement data via Wireless Wildlife. One individual (LPD006) was found to have died for unknown causes in February 2015 after only 88 days. The transmitter was redeployed on a new leopard tortoise individual.

### Climatic variables

Hourly temperature and rainfall data were collected from the South African Weather Service (Pretoria, South Africa) [64], using Beaufort-West weather station (station number: 0092081 5), approximately 45 km west of study sites. Data were collected from September 1993 to end of study period (December 2015) to compare study period to previous years. Mean temperature and total rainfall (mm) were collected for three temporal scales for the study period: bihourly and daily for movement analysis, and monthly for long-term data comparisons.

### Data screening

Screening of data were carried out to discard incorrect location fixes using ‘adehabitatLT’ version 0.3.20, ‘adehabitatMA’ version 0.3.10, ‘ade4’ version 1.7–4 and ‘sp’ version 1.2–3 in R version 3.1.2 [65, 66], using RStudio version 0.98.1091 [67]. Data were discarded based on values for extreme horizontal dilution of precision (HDOP) values, incorrect time zones, incomplete or dubious transmitter data (e.g. negative activity), impossible and improbable movement distances, and z-coordinate error.

### Habitat extraction and proximity

A 2014 South Africa land cover layer was downloaded from GEOTERRAIMAGE (Pretoria, South Africa). The land cover layer is a raster that categorises land area as a habitat; for example, grassland, low shrubland, or cultivated commercial fields. ArcGIS was used to crop raster to local area. Habitats were extracted from the raster layer to each GPS location, with extracted results saved as an Excel file. The land cover raster layer was converted to place a point for each 3 m × 3 m pixel.

In addition, two other important layers were also used; inland water areas, and manmade water source points; taken from a 1:50,000 topographical map of South Africa. These two layers represent potentially important water features that may not be recognised by the land cover layer, as the feature is within a forested area (and so would be classified as the top layer) or is too small to be recognised in the South Africa land cover layer (manmade wells and feeding or drinking stations for livestock). We calculated an individual’s Euclidean distance to water resources (inland water areas and manmade water source points) to create an additional predictor variable for generalized linear mixed models (GLMMs).

For purpose of identifying associations with habitats that might supply more food resources, we grouped other habitat categories (dense bush, open bush, wetland, grassland, cultivated commercial fields) based on expectations compared to low shrubland and non-vegetated habitats (Table 1). We grouped the cultivated commercial field categories, which were previously separated into high, medium or low layers. Other habitat types were excluded, due to no nearby tortoise location data. We used the proximity function to also calculate distances to a) water resources, b) increased food resources, and 3) cultivation areas.

**Table 1:** Habitat and resource groupings used in the current study

Land cover layer classification	Updated classification	Additional layers	Resource categories
Water seasonal	Water seasonal	-	Water
Water permanent	Water permanent	-	Water
Wetlands	Wetlands	-	Food
Thicket/Dense bush	Dense bush	-	Food
Woodlan/Open bush	Open bush	-	Food
Grassland	Grassland	-	Food
Low shrubland	Low shrubland	-	None
Cultivated comm fields (high)	Cultivated commercial fields	-	Food
Cultivated comm fields (med)	Cultivated commercial fields	-	Food
Cultivated comm fields (low)	Cultivated commercial fields	-	Food
Bare none vegetated	Non-vegetated	-	None
-	-	Manmade water source points	Water
-	-	Inland water areas	Water

(Habitat classifications were from 2014 South Africa land cover layer, GEOTERRAIMAGE (Pretoria, South Africa). Additional layers include manmade water source points and inland water areas from a 1:50,000 topographical map of South Africa. Resource categories are based on expected access to increased food resources and water.)

### Statistics

Prior to calculating distances between tortoise locations, transmitter fix error was quantified. We used Euclidean distances between fix locations and known transmitter locations in Pietermaritzburg, South Africa, prior to transmitter deployment. Test data had a mean (± SE) fix error of 17.01 ± 0.59 m (range: 1.78 to 134.78 m).

Distances between transmitter locations and subsequent statistical analyses were carried out in R [65] using RStudio [67]. Bihourly movement was calculated using ‘adehabitatLT’, ‘adehabitatMA’, ‘ade4’ and ‘sp’ [66]. We assumed each movement was Euclidean distance between successive locations [68]. We assumed each location fix was affected by a fix error. We ranked calculated distance for each movement and assumed larger distances were more likely to be due to larger fix errors. Therefore, we corrected each calculated distance by deducting inverse log of the quantile for the known error fixes (Equation 1), where d_rank_ is the d_th_ percentile from log transformed known error distribution, d_est_ is estimated distance between points, and d_corr_ is corrected distance.

1 $$ {d}_{corr}={d}_{est}-{10}^{\log \left({d}_{rank}\right)} $$

In addition to the above, data was also screened based on z-coordinate error [69]. Internal transmitter altitude estimates were compared with approximate heights in digital elevation models (DEMs)—freely available from ‘raster’ version 2.5–2 package [70]—and discarded when z-coordinate error exceeded 100 m. Fixes were also discarded if time record was not approximate to predefined settings (e.g. > 120 s after intended fix), which would indicate error in transmitter functionality or inaccuracy based on receiving satellite data.

Cumulative distances were calculated for daily and monthly periods for all but one individual: LPD006 was excluded from analyses due to death and reduced data. Bihourly and daily movement distances were tested for normality using a ‘quantile-quantile’ plot using ‘stats’ version 3.1.2 package in R [65]. As these data were heavily right-skewed, log transformations of both bihourly and daily datasets were carried out prior to analyses. As tortoise movement can be strongly affected by environmental conditions [22], we compared the study year to long-term data for the region. We used Welch two sample t-tests to compare monthly mean temperature and total rainfall data to previous years.

GLMMs were used to create and test models to compare effect of predictor variables on bihourly and daily movement. Predictor variables used were a mix of individual, environmental and weather variables; habitat, month, sex, time of day, distance from water source, mean temperature, and total rainfall. Tortoise ID was set as the random variable to account for pseudoreplication. To ensure data were standardised, we used the standardize function in ‘arm’ version 1.8–6 package in R [71]. For daily models, habitat type for each datapoint was determined as the most common habitat type used by individual for each day. Time of day was not included in daily analysis, as hour-sensitive data were combined for each day. For the continuous predictor variables in daily models we took the mean result for all locations during that day. Aside from temperature, continuous predictor variables used in bihourly models did not use mean results. All possible combination models were tested using the ‘glmer’ function within ‘lme4’ version 1.1–10 package [72] and ‘dredge’ function using ‘MuMIn’ version 1.15.6 package [73].

Top candidate models (ΔAIC_c_ < 2) were selected for bihourly and daily GLMMs, with models ranked based on values for AIC_c_; Akaike’s Information Criterion, adjusted for small sample size [74]. As both GLMMs provided more than one top model, model averaging was used to identify important predictor variables and model coefficients based on those variables. All mean movement results are reported with standard error (± SE). Interaction effects for important predictor variables in both models were tested using analysis of deviance in ‘phia’ version 0.2–1 package [75]. For bihourly models, we tested effect of month and time on other variables, whilst month and habitat were tested for daily models. Predictor variables not identified as important were excluded from post-hoc analyses.

## Results

### Movement summary

As mentioned, relocation data were collected from 10 telemetry transmitters on adult leopard tortoises from November 2014 to December 2015. LPD048 was tracked for only 283 days, as the transmitter was redeployed following death of LPD006. All other individuals were tracked for a minimum of 359 days. In total, 42,467 data points were collected (Table 2). The data screening process removed 5,413 data points: a mean (± SE) of 541.3 (± 77.20) per individual. The final bihourly dataset consisted of 37,054 data points.

**Table 2:** Biological information for each telemetered individual leopard tortoise, along with the number of geolocation fixes used in final analyses for each

Individual	Farm	Sex	Body mass (g)	Screened fixes
LPD001	Baakensrug	Female	11,685	4017
LPD002	Baakensrug	Female	11,580	3587
LPD004	Baakensrug	Male	7,425	4122
LPD010	Kamferskraal	Female	26,167	4159
LPD011	Kamferskraal	Female	18,400	3647
LPD013	Kamferskraal	Male	12,560	3790
LPD015	Elandsfontein	Male	15,125	3941
LPD016	Elandsfontein	Male	14,870	3330
LPD017	Elandsfontein	Female	16,638	3884
LPD048*	Baakensrug	Male	9,275	2577

*Telemetered individual LPD006 was found dead through course of study. The GPS unit was recovered and reattached to a new individual (LPD048). Data from the dead tortoise were excluded from the analyses

**Fig. 2:**
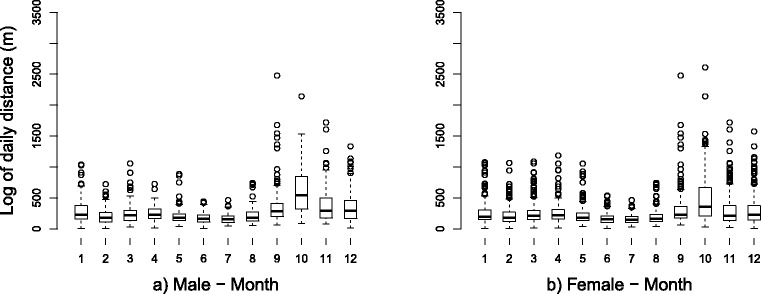
Sex variation in daily movement. Daily movement in adult **a**) male (*n* = 5), and **b**) female (*n* = 5) leopard tortoises for each month of the study period, near Beaufort West, South Africa. (Numbers on the x-axis correspond to months; e.g. 1 = January, 2 = February, 3 = March, etc)

Bihourly and daily movement of leopard tortoises were calculated for each individual throughout course of the study period. Overall mean distance moved by leopard tortoises was 257.7 (± 3.64) m per day (range: 1.79 to 2611.24 m). Males (291.6 ± 6.00 m) appeared to move further than females (225.9 ± 4.11 m), although largest daily distance moved was by a female (2611.24 m). The largest daily distance by a male tortoise was 2477.31 m. Movement varied seasonally, with spring months of September (302.0 ± 14.68 m), October (471.7 ± 20.57 m), and November (295.6 ± 14.66 m) showing largest daily movement distances (Fig. 2, Table 3). Mean daily movement was consistently above 150 m per day throughout much of the year, but winter months showed the shortest movement distances; June (162.1 ± 4.84 m), July (157.6 ± 4.09 m), and August (191.1 ± 6.46 m).

**Table 3:** Sex differences in daily movement of leopard tortoises for each month, along with weather conditions

Month	Daily distance moved (mean ± SE) in metres			Average temperature (°C)	Total Rainfall (mm)
	Total	Male	Female		
January	256.0 ± 11.29	292.2 ± 18.99	227.5 ± 13.16	25.3	7.0
February	218.0 ± 9.71	200.9 ± 12.97	232.3 ± 14.08	22.7	13.2
March	248.2 ± 9.10	240.7 ± 12.40	255.6 ± 13.31	22.2	28.4
April	256.5 ± 9.59	291.7 ± 6.00	260.1 ± 16.05	16.8	0.0
May	218.2 ± 8.15	208.5 ± 10.14	227.8 ± 12.74	16.6	1.2
June	162.1 ± 4.84	175.4 ± 7.08	148.9 ± 6.44	11.5	15.2
July	157.6 ± 4.09	165.1 ± 6.23	150.0 ± 5.24	10.6	20.4
August	191.1 ± 6.46	212.2 ± 10.30	170.5 ± 7.54	14.7	25.0
September	302.0 ± 14.68	371.1 ± 26.15	233.0 ± 10.80	15.1	8.8
October	471.7 ± 20.57	624.1 ± 29.74	319.3 ± 22.64	20.7	17.8
November	296.6 ± 14.66	390.1 ± 24.41	207.2 ± 13.21	19.9	10.2
December	306.7 ± 14.31	349.8 ± 23.02	275.4 ± 17.89	23.3	23.8

(Weather data supplied by South African Weather Service (Pretoria, South Africa) for Beaufort West area, South Africa)

### Habitat type associations

Habitat extractions showed variability between individual leopard tortoises. Whilst 85.1% of all data points were within habitat classified as ‘low shrubland’, two individuals were found in low shrubland habitat less than 50% of the time. In each, dense bush was an important habitat type, with over 30% of data points. Use of wetlands (0.05%), grassland (1.1%), and non-vegetated (2.1%) habitats were used infrequently, although amount of land covered by each of these was much lower than low shrubland. There were changes to habitat use throughout the year (Table 4), in particular during winter months (June to August), where individuals appeared to stay in low shrubland areas.

**Table 4:** Leopard tortoise habitat types used throughout the year

Habitat type	Jan	Feb	Mar	Apr	May	Jun	Jul	Aug	Sep	Oct	Nov	Dec	Mean (± SE)
Low shrubland	2351	1999	2312	2241	2900	3019	3156	2963	2672	2634	2187	2659	2591.08 ± 107.34
Non-vegetated	158	140	86	8	34	18	15	50	80	42	25	129	65.42 ± 15.15
Dense bush	203	340	453	137	195	7	14	194	264	321	788	144	255.00 ± 60.98
Open bush	120	4	102	191	67	41	20	19	115	174	121	31	83.75 ± 18.08
Grassland	2	1	36	1	25	0	0	0	64	55	41	163	32.33 ± 13.69
Wetlands	3	6	5	0	0	0	0	0	0	2	1	0	1.42 ± 0.62
Cultivated commercial fields	0	31	127	3	0	0	0	0	0	40	0	0	16.75 ± 10.78
Water permanent	0	0	6	0	0	0	0	0	0	0	0	0	0.50 ± 0.50
Total	2837	2521	3127	2581	3221	3085	3205	3226	3195	3268	3163	3126	

(Numbers represent the number of data points for each habitat type for each month of the year.)

The above is also reflected by associations leopard tortoises had with features. Only one telemetered individual (LPD011) approached within 250 m of cultivated commercial fields. Majority of data points showed no association with water resources, with 77.2% of data points away (>250 m) from these areas. Only 47.2% of data points were within close proximities to habitats listed as providing increased food resources.

### Weather comparison to previous years

Mean monthly temperature during study period (18.2 ± 1.36 °C) did not significantly deviate from long-term (from September 1993) monthly temperature (17.9 ± 0.26 °C) (Welch two sample *t*-test, t_(12)_ =−0.2096, *P* = 0.838). Similarly, mean monthly rainfall was low (14.2 ± 2.65 mm) when compared with other years (21.5 ± 1.43 mm), though no significant difference was found (t_(12)_ = 0.4005, *P* = 0.696).

### Bihourly movement

**Fig. 3:**
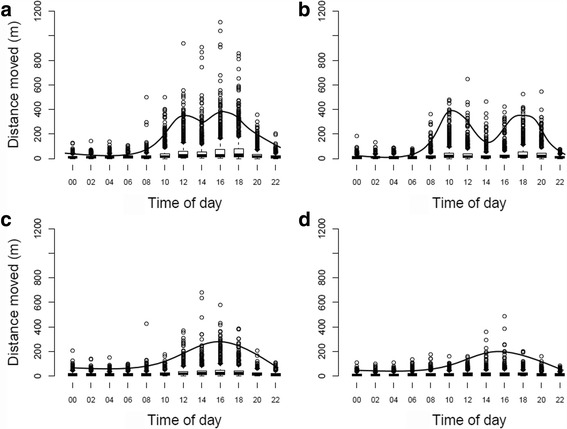
Seasonal variation in daily movement. Bihourly movement of adult leopard tortoises (*n* = 10) throughout day and night in **a**) spring (September to November), **b**) summer (December to February), **c**) autumn (March to May), and **d**) winter (June to August), near Beaufort West, South Africa. Lines indicate general activity patterns for that season

Bihourly movement behaviour of leopard tortoises showed a bimodal pattern during spring and summer, with highest movement during late morning and mid-afternoon. This bimodal pattern was more pronounced in summer (Fig. 3), whereby movement was highest around 10:00 and 18:00 and generally decreased at 14:00. A unimodal pattern is observed during autumn and winter. Movement was identified during night-time hours during all months of the year, though this was decreased in winter.

A total of 128 candidate models were tested to predict bihourly movement of leopard tortoises. We identified two top candidate models (ΔAIC_c_ < 2) (Table 5). Model averaging highlighted five important predictor variables, based on relative importance (RI); month, time of day, distance from water source, mean temperature (all RI = 1.00), and total rainfall (RI = 0.44) (Table 6). Habitat type and sex were not statistically significant predictor variables in either top candidate model.

**Table 5:** Bihourly movement top models in the current study showing results from top GLMMs comparing model fitness for bihourly movement for leopard tortoises

Model	df	log.like	AIC_c_	ΔAIC_c_	*w*_*i*_
**month** + **time** + **water** + **temp**	**27**	−**23481.89**	**47017.82**	**0**	**0.521**
**month** + **time** + **water** + **rain** + **temp**	**28**	−**23481.13**	**47018.30**	**0.48**	**0.409**
month + sex + time + water + temp	28	−23483.52	47023.10	5.28	0.037
month + sex + time + water + rain + temp	29	−23482.77	47023.58	5.76	0.029
habitat + month + time + water + temp	34	−23480.30	47028.67	10.86	0.002
habitat + month + time + water + rain + temp	35	−23479.79	47029.65	11.83	0.001

Notes: *df* degrees of freedom, log.like = log likelihood, ΔAIC_c_ = deviation for AIC_c_ compared with top model, *w*_*i*_ = AIC_c_ weight

(Predictor variables included habitat type, month, sex, time of day, distance from water source, mean temperature, and total rainfall. Rows shown in bold indicate top models (ΔAIC_c_ < 2). Rainfall and temperature measurements were from the two hour period prior to positional fix, using data supplied by South African Weather Service (Pretoria, South Africa) for Beaufort West area, South Africa)

**Table 6:** Statistically significant predictor variables for bihourly movement in leopard tortoises

	β	SE	*z*	Confidence intervals		RI
				2.5%	97.5%	
(Intercept)	1.009	0.037	27.26	0.94	1.08	-
Month^a^						1.00
January	−0.010	0.013	0.72	−0.04	0.02	
February	−0.047	0.013	3.47	−0.07	−0.02	
March	−0.018	0.013	1.46	−0.04	0.00	
May	−0.025	0.012	2.02	−0.05	−0.00	
June	−0.086	0.013	6.76	−0.11	−0.06	
July	−0.081	0.013	6.34	−0.11	−0.06	
August	−0.057	0.037	4.66	−0.08	−0.03	
September	0.044	0.012	3.55	0.02	0.07	
October	0.0114	0.012	9.25	0.09	0.14	
November	0.010	0.012	0.79	−0.01	0.03	
December	−0.011	0.013	0.87	−0.04	0.01	
Time of day^b^						1.00
2 am	−0.008	0.012	0.68	−0.03	0.02	
4 am	0.015	0.012	1.27	−0.01	0.04	
6 am	0.021	0.012	1.77	−0.00	0.04	
8 am	0.058	0.012	4.86	0.03	0.08	
10 am	0.176	0.012	14.90	0.15	0.20	
12 pm	0.228	0.012	18.90	0.20	0.25	
2 pm	0.190	0.012	15.21	0.17	0.21	
4 pm	0.248	0.013	19.41	0.22	0.27	
6 pm	0.281	0.012	22.53	0.26	0.31	
8 pm	0.143	0.012	11.93	0.12	0.17	
10 pm	0.018	0.012	1.52	−0.01	0.04	
Distance from water	−0.101	0.008	11.86	−0.12	−0.08	1.00
Rainfall	0.016	0.005	0.82	0.01	0.03	0.44
Temperature	0.072	0.008	8.48	0.06	0.09	1.00

Notes: ^a^ = April used as reference for month variable. ^b^ = 00 am used as reference for time of day variable

(Unconditional parameter estimates, standard error, confidence intervals and relative importance (RI) of tested predictor variables for bihourly displacement distances, using two top candidate models (ΔAIC_c_ < 2). Predictor variables shown include month, time of day, distance from water source, total rainfall, and mean temperature)

**Fig. 4:**
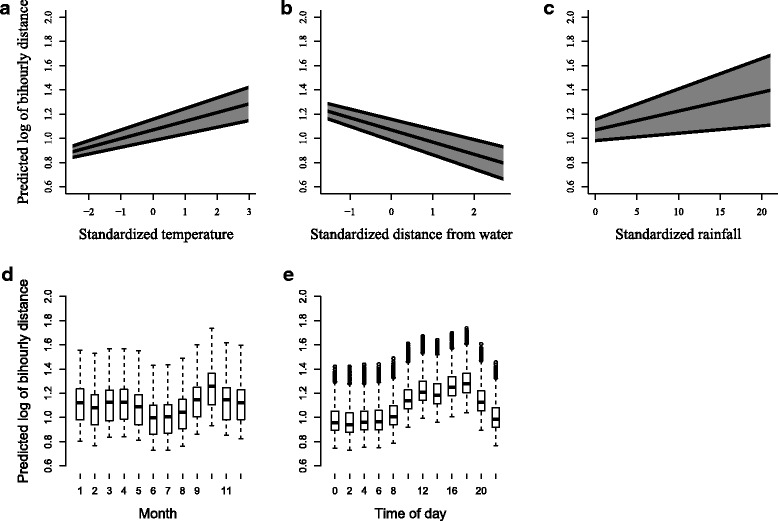
Variables predicting bihourly movement. Bihourly movement for leopard tortoises, Central Karoo, South Africa, as predicted by model averaging using two top candidate generalized linear mixed models. Predictor variables with relative importance (RI) include **a**) standardized mean temperature (RI = 1.00), **b**) standardized distance from water source (RI = 1.00), **c**) standardized rainfall (RI = 0.44), **d**) month (RI = 1.00), and **e**) time of day (RI = 1.00). For month, 1 = January, 2 = February, 3 = March, etc

Results showed a positive relationship between movement distance of leopard tortoises and mean temperature, and rainfall (Fig. 4). There was a negative relationship for movement with distance from water source. Month as a predictor variable also showed that movement was expected to be highest in spring (September to November), with lowest movement predicted in winter (June to August).

A significant interactive effect was found for month and distance from water, indicating that effect of distance from water on bihourly movement is dependent on time of year (Table 7). No other interaction effects were significant.

**Table 7:** Analysis of deviance table for predictor variables of bihourly movement

Predictor variables	LR	df	Probability
Time of day	25962.2	11	*P* > 0.001
Month	11721.4	11	*P* > 0.001
Temperature	1496.8	1	*P* > 0.001
Rainfall	32.5	1	*P* > 0.001
Distance from water	1703.6	1	*P* > 0.001
Month : Temperature	11.6	11	*P* = 0.393
Month : Rainfall	1.9	10	*P* = 0.997
Month : Distance from water	731.2	11	*P* > 0.001
Time of day : Month	39.4	121	*P* = 1.000
Time of day : Temperature	2.3	11	*P* = 0.997
Time of day : Rainfall	1.2	11	*P* = 1.000
Time of day : Distance from water	10.6	11	*P* = 0.474
Time : Month : Temperature	19.4	121	*P* = 1.000
Time : Month : Rainfall	6.8	49	*P* = 1.000
Time : Month : Distance from water	46.7	121	*P* = 1.000

Interactive effect of statistically significant predictor variables for predicting bihourly movement in leopard tortoises. Predictor variables are shown alone, and with potential interactive variables, along with likelihood ratio (LR) chi-squared statistic, degrees of freedom (df) and statistical significance (P) values

### Daily movement

When aggregating daily habitat type, only one location recorded wetlands as a habitat. This record was excluded from the dataset prior to GLMM analysis. A total of 64 candidate models were tested to predict daily movement distances. We identified two top candidate models (ΔAIC_c_ < 2) (Table 8). The important predictor variables were habitat type, month, distance from water source (all RI = 1.00), and mean temperature (RI = 0.70) (Table 9).

**Table 8:** Daily movement top models

Model	df	log.like	AIC_c_	ΔAIC_c_	*w*_*i*_
**habitat** + **month** + **temp** + **water**	21	−429.80	901.87	0	0.653
**habitat** + **month** + **water**	20	−431.64	903.52	1.65	0.286
habitat + month + sex + temp + water	22	−431.70	907.69	5.83	0.035
habitat + month + sex + water	21	−433.54	909.33	7.47	0.016
habitat + month + temp + water + rain	22	−433.26	910.80	8.93	0.007
habitat + month + water + rain	21	−435.33	912.93	11.06	0.003

Notes: *df* degrees of freedom, log.like = log likelihood, ΔAIC_c_ = deviation for AIC_c_ compared with top model, *w*_*i*_ = AIC_c_ weight

Results from top GLMMs comparing model fitness for daily movement for Leopard Tortoises. Predictor variables included habitat type, month, sex, distance from water source, mean temperature, and total rainfall. Rows shown in bold indicate top models (ΔAIC_c_ < 2). Rainfall and temperature measurements were provided by South African Weather Service (Pretoria, South Africa) for Beaufort West area, South Africa

**Table 9:** Statistically significant predictor variables for daily movement

	β	SE	*z*	Confidence intervals		RI
				2.5%	97.5%	
(Intercept)	2.345	0.03	73.81	2.28	2.41	-
Habitat type^a^						1.00
Non-vegetated	−0.291	0.04	7.75	−0.36	−0.22	
Dense bush	−0.138	0.02	6.81	−0.18	−0.10	
Open bush	−0.137	0.03	4.15	−0.20	−0.07	
Grassland	−0.119	0.05	2.39	−0.22	−0.02	
Cultivated fields	−0.132	0.06	2.05	−0.26	−0.01	
Month^b^						1.00
January	−0.010	0.03	0.34	−0.07	0.05	
February	−0.077	0.03	2.81	−0.13	−0.02	
March	0.005	0.03	0.20	−0.05	0.06	
May	−0.056	0.02	2.48	−0.10	−0.01	
June	−0.170	0.03	6.63	−0.22	−0.12	
July	−0.172	0.03	6.55	−0.22	−0.12	
August	−0.100	0.02	4.31	−0.15	−0.05	
September	0.087	0.02	3.76	0.04	0.13	
October	0.207	0.02	8.57	0.16	0.25	
November	0.027	0.02	1.14	−0.02	0.07	
December	0.014	0.03	0.54	−0.04	0.07	
Temperature	0.048	0.01	3.20	0.02	0.08	0.70
Distance from water	−0.147	0.02	8.86	−0.18	−0.11	1.00

Notes: ^a^ = Low shrubland used as reference for habitat type variable. ^b^ = April used as reference for month variable

Unconditional parameter estimates, standard error, confidence intervals and relative importance (RI) of tested predictor variables for daily movement, using two top candidate models (ΔAIC_c_ < 2). Predictor variables shown include most common habitat type, month, mean temperature, and distance from water source

**Fig. 5:**
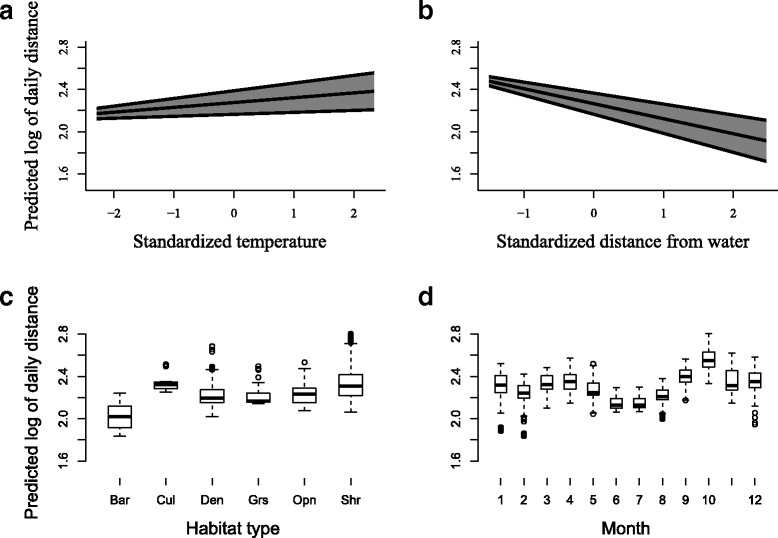
Variables predicting daily movement. Daily movement for leopard tortoises, Central Karoo, South Africa, as predicted by model averaging using two top candidate generalized linear mixed models. Predictor variables with relative importance (RI) include **a**) standardized mean temperature (RI = 0.70), **b**) standardized distance from water source (RI = 1.00), **c**) habitat type (RI = 1.00), and **d**) month (RI = 1.00). Abbreviations include: Bar = non-vegetated, Cul = cultivated fields, Den = dense bush, Grs = grassland, Opn = open bush, and Shr = low shrubland. Spr = spring, Sum = summer, Aut = autumn, and Win = winter. For month, 1 = January, 2 = February, 3 = March, etc

Temperature (positive relationships), distance from water source (negative relationship), and month variables presented similar results when compared with bihourly models (Fig. 5). Effect of habitat type on predicted movement was variable. Highest movement was predicted at low shrubland and cultivated commercial fields, whilst non-vegetated land predicted lowest movement. Sex and rainfall were not statistically significant predictor variables in either top candidate model predicting daily movement.

Significant combination effects for daily movement were shown for month, habitat type, and distance from water, indicating that effect of these variables on daily movement is affected by each other (Table 10). Temperature did not show any interactive effects with other important predictor variables.

**Table 10:** Analysis of deviance table for predictor variables of daily movement

Predictor variables	LR	df	Probability
Month	8292.0	11	*P* < 0.001
Habitat	3938.9	5	*P* < 0.001
Temperature	98.6	1	*P* < 0.001
Distance from water	810.5	1	*P* < 0.001
Month : Habitat	120.9	29	*P* < 0.001
Month : Temperature	3.0	11	*P* < 0.001
Month : Distance from water	126.4	11	*P* < 0.001
Habitat : Temperature	0.4	5	*P* = 0.990
Habitat : Distance from water	14.7	5	*P* = 0.012
Month : Habitat : Temperature	2.9	26	*P* = 1.000
Month : Habitat : Distance from water	136.3	26	*P* < 0.001

Interactive effect of statistically significant predictor variables for predicting daily movement in leopard tortoises. Predictor variables are shown alone, and with potential interactive variables, along with likelihood ratio (LR) chi-squared statistic, degrees of freedom (df) and statistical significance (P) values

## Discussion

Movement and activity in tortoises is influenced by life history, resource availability, thermoregulatory necessities, habitat fragmentation, and reproductive requirements [76]. Although daily movement in leopard tortoises is generally affected by season, daily movement is generally short. Previous estimations of daily movement of leopard tortoises (usually < 100 m) [31, 33, 43] were much lower than present study (256.97 ± 3.56 m per day). Sporadic large movements by individuals (up to 8 km) have been recorded [33], although most other studies show maximum long distance movement of leopard tortoises is approximately 4 km [26, 31]. Movement in more arid environments of the Nama-Karoo [33] was higher than in Eastern Cape, South Africa [43], Swaziland [31], and Zimbabwe [42]. Variation in movement distances of the above studies has been attributed to seasonal temperature, availability of food resources, rainfall, differences in mean body mass, and need to ingest key isolated resources (e.g. sodium) [31, 33, 42]. In our study GLMMs identified multiple important climatic, environmental, and individual predictor variables on two temporal scales (bihourly and daily). Three variables (mean temperature, distance from water resource, and month) were important predictor variables in top candidate models for both GLMMs. Three additional predictor variables were also important: rainfall and time of day (bihourly movement), and habitat type (daily movement).

Male leopard tortoises moved further than females overall, and in seven individual months, including each month in spring (September to November), which is when breeding activity (reproduction and egg-laying) in leopard tortoises is typically high [46, 47]. However, sexual differences in movement were not highlighted in either GLMM. This is contradictory to the majority of published tortoise movement ecology studies which show that male movement is significantly higher than females [22, 32, 36, 37]. Peak movement in leopard tortoises of both sexes occurred in spring (September to November). There was a female lag behind males for peak movement: male movement began to increase in September, whilst female movement increased in October. October was the peak month of movement for both sexes. This supports previous research on leopard tortoises [58]. These peaks could be related to individual reproductive status. Mate-searching in tortoises, conducted primarily by males, generally occurs in spring when resource availability and climatic conditions are suitable [58]. Female movement may increase following fertilisation in mid-spring, as search for suitable egg-laying habitat begins [36]. As leopard tortoises can occur at very low densities (e.g. 0.017 tortoises per ha) in some parts of the Karoo [25], it can be expected that males make much larger movements to find mates compared with other species and other regions. This is supported by research on *Gopherus* tortoises, where males made larger daily spring movements (up to 500 m) in areas of lower burrow (and therefore population) density in search of mates [37]. Associated with reproduction is an increase in energy costs: especially for females with regards to producing eggs [32, 35]. Tortoises of both sexes generally increase activity, not only to search for mates and egg-laying habitat, but also for increased demand for food intake and, in case of females, other important resources [35, 37]. No specific instances of reproductive behaviour was observed, though one male (LPD013) was observed alongside several non-telemetered females at a watering point during December 2015.

Habitat type was found to be an important predictor variable for predicting daily leopard tortoise movement. Daily movement was shown to be highest in low shrubland habitat, the most-used habitat type. Cultivated commercial fields also predicted high movement distances, although only one individual used this habitat. We classified multiple habitat types as providing an expected higher supply of food resources, compared with low shrubland and non-vegetated habitats. However, only two individuals remained in these areas throughout majority of the study. The results showed that non-vegetated habitat type was predicted to have lowest movements by daily models, which supports previous research that shows that activity is decreased when resources are low [22]. Our classification for higher food resources was based on expected resources from a land cover layer. However, no surveys were conducted for these habitat types and diet in leopard tortoises is extremely adaptable. Diet-switching behaviour has been identified in leopard tortoises whereby they feed on different plants through year, depending on resource availability [18]. In addition, they will feed on a wide variety of foods, including grasses, forbs, fruits, and succulents [18]. Succulents are even avoided by livestock [18], and are sometimes present in over-grazed areas, such as non-vegetated habitat (unpublished observations). Therefore, smaller movements by individuals in non-vegetated habitat may be due to a higher food searching efficiency by leopard tortoises.

Distance from known water sources was an important predictor in both GLMMs. Contrary to our predictions, movement decreased as individuals moved away from water resources. As forbs (74.5%) and succulents (51.0%) generally represent a large percentage of their diet [77], it is likely that high water content of these plants could supplement water intake for individuals for much of the year, especially in such a water scarce habitat [31]. In addition, leopard tortoises are able to adapt digestive parameters (food intake, water loss and urine osmolality) in response to diet to maintain body mass and water balance [20]. This could make them even more resilient to lack of water associated with arid environments [22, 24]. Despite their ability to obtain much of their water requirements from food intake and metabolic water, they may need to drink free standing water so supplement their water budget demands and restore osmotic homoeostasis, as high electrolyte contents can cause severe stress and sometimes death [22–24].

It appears leopard tortoise movement increased when individuals were closer to water resources, perhaps because of knowledge of resource localities: animals maintain and continually update a cognitive map [78]. Whereas tortoises further away from permanent water appear to rely on food resources for water intake, if known water sources exist within an animal’s home range, individuals may make regular movements to maintain internal water balance, though water balance was not measured. Most telemetered individuals had little or no association with known water resources. However, many non-telemetered individuals were frequently observed congregated around manmade watering points and dams (unpublished observations). Such observations have been previously reported, whereby home range of several individuals overlapped at manmade water sources [25, 26]. This presents a potential issue, considering the upcoming introduction of fracking activities in the Karoo (expected before end of 2017) [8, 9, 79, 80], as contamination of these water sources through increased water salinity and decreased water quality [10, 11] could adversely affect a large number of individuals that rely on these permanent water sources. Demand for water in the region already exceeds availability [81, 82], with demand projected to increase by up to 150% by 2025 [79]. Up to 90% of water use in South Africa is supplied from surface resources [82], yet infrequent rains in the Karoo rarely reach rivers and cannot supply demand [79]. Whilst it appears that tortoises are able to use food sources for water, it is unknown how fracking will impact these food sources. Further research is required to assess how fracking will affect local human, animal, and plant communities.

The adaptations to water scarce environments are especially important due to unpredictable and infrequent nature of rainfall in the Karoo [49, 50]. Increased tortoise activity is usually found to be associated with rainfall [22, 27–29], with several species having physiological and behavioural adaptations to facilitate drinking rainwater [47, 83]. Our results support these previous findings, with bihourly movement showing a positive relationship with rainfall. This is in contrast to lack of correlation between activity and rainfall found by McMaster and Downs [58] in a similar region. However, one must be cautious when interpreting our results. Whilst no significant difference was found between monthly rainfall during the study year and previous years, rainfall was lower. The mean daily rainfall was 0.44 mm, although over half of rainfall days yielded less than 2 mm of rain. Rainfall also did not fall in any one particular season; 12 days in spring, 15 days in summer, 6 days in autumn, and 20 days in winter. Tortoises have the ability to use their bladders as water reservoirs [23]. As such, early rains may be more important, and could explain why rainfall was not shown as an important predictor variable in daily models. Such unpredictability in rainfall increases importance of permanent water resources. Movement studies should ideally be conducted over several seasons, though financial, battery life, and time restrictions vary.

Whilst rainfall is unpredictable, temperature is less so, and has been previously shown as important in dictating movement in tortoise studies [30, 31]. Tortoises are ectothermic, and so activity is directly related to local environmental conditions to support metabolism [58]. As such, tortoises generally move more in spring and summer, with movement decreased in winter [31–34], though patterns are likely more complex and related to specific environments and climatic conditions. Behaviour is also important: tortoises bask in morning sun prior to becoming active during the day [58]. Temperature and month were important predictor variables in both GLMMs. Our bihourly data also showed a basic bimodal movement pattern in warmer seasons, when maximum daily temperatures frequently exceeded 30 °C. This bimodal activity pattern (with movement higher during mornings and evenings) is a behavioural adaptation that allows individuals to avoid extreme temperatures, which may cause severe stress or death [26, 28, 36, 58, 59]. Indeed, hours of activity restriction due to increased temperatures associated with global warming is believed to be a main predictor for local extinctions of yellow-footed tortoises, *Chelonoidis denticulata* [84]. Some species (e.g. *Testudo* spp.) reduce activity in summertime to avoid extreme temperatures [30], whilst others (e.g. *Gopherus* spp., African spurred tortoise, *Centrochelys sulcata*) remain in burrows over many weeks [47]. Leopard tortoises are not known to dig burrows, but will use shade of bushes and boulders to shield themselves from sun [58, 85].

Due to the close relationship between temperature and activity, leopard tortoise movement is generally restricted in cooler temperatures, such as during winter months and during night-time hours. In more moderate climates, tortoises brumate to avoid cold conditions [22, 30, 34, 47]. However, mean winter (June to August) temperatures in the Karoo are still warm enough to facilitate movement: over one third of winter days had maximum temperatures exceeding 20 °C. Mean daily movement of leopard tortoises during winter months exceeded 150 m. Leopard tortoises do not typically brumate [26, 31], although isolated records do occur [25]. In contrast to bimodal activity patterns in warmer months, a unimodal activity pattern was observed in autumn and winter, as described previously by McMaster and Downs [58]. McMaster and Downs [58] also noted leopard tortoises are generally inactive during night-time. However, our results show night-time movement does occur, especially in summer and autumn months. Night-time foraging in leopard tortoises has been reported in one individual previously [46]. It is currently unknown what may facilitate night-time movement, although it appears that night-time temperatures are often non-restrictive during these periods. More research is required to ascertain variables enabling this night-time movement. Other potentially important variables, such as environmental illumination, may also affect movement ability during night-time hours when temperatures are non-restrictive.

Information regarding drivers of movement, and periods in which movement is highest, can be used to mitigate against other threats to tortoises. For example, electric fencing is used in much of the Karoo as a means to control predation on livestock by caracal and black-backed jackal [41]. This electric fencing causes mortalities in a number of mammals, reptiles, and amphibians [56], though fatalities are highest with respect to tortoise species and ground pangolin (*Smutsia temmincki*) [56]. Leopard tortoises account for most (>86%) electric fencing related reptile mortalities [56, 57], likely related to their size and spatial ecology. As electric fencing is becoming more affordable in South Africa, tortoise mortalities by electrocutions is increasing. Whilst it has been recommended that raising the electric line to a minimum height of 250 mm could reduce mortalities [57], strategic planning can also be incorporated into operations by reducing use of electric fencing when and where tortoises are most active: in mornings and evenings, mating season, and nearer to water sources.

## Conclusions

Our results further display the relationship between water as a resource and movement in leopard tortoises. We provide evidence individuals can use either one of two basic movement behaviours in relation to water sources in water scarce environments: either an individual’s home range and movement is such that it includes permanent water resources, allowing regular long-distance movements to replenish water storage; or access to these resources are excluded and there is instead a reliance on food resources (such as grasses, forbs, and succulents) as the primary source of water. It is known from previous research that multiple tortoise species are able to tolerate high electrolyte concentrations, though drinking water is a requirement for urination and restoration of water balance. Further research should be carried out on potential impacts of fracking activities, as contamination and increased salination of groundwater may affect ability to restore water balance. Subsequent dehydration could cause severe stress and possible mortality.

In particular our research identified temporal and spatial conditions in which leopard tortoise movement increased. Such information can be used to guide designs, constructions and operations of electric fencing. As leopard tortoise movement is higher in areas closer to water resources, we advise that electric fencing does not occur within close proximities to these areas. We also advise that electric fencing should not operate during spring and summer months, whereby reproductive and general activities are increased. However, our data shows tortoises move throughout the year, and even during night-time hours. Whilst is may not be possible to avoid all mortalities related to electric fencing, we hope that the above suggestions could reduce impacts. Increasing time between shocks, or alternating in electric fence functionality at intervals may also enable shocked individuals to escape should contact occur. We also support previous suggestions whereby the electrified line is raised to a minimum height of 250 mm.

## Abbreviations

AIC: Akaike’s information criterion
GLMM: Generalized linear mixed model
GPS: Global positioning system
GSM: Global system for mobile communications
HDOP: Horizontal dilution of precision
RI: Relative importance
UHF: Ultra high frequency
